# Supplementary material for: Inverse association of dietary consumption of n3 and n6 fatty acids with hyperuricemia among adults
Source: PLoS One. 2025 Mar 13;20(3):e0317490. doi: 10.1371/journal.pone.0317490 (PMC11906074; doi:10.1371/journal.pone.0317490)
Supplement: Table S1 — (DOCX) [file pone.0317490.s001.docx]

**Supplementary Table 1** The ORs (95% CIs) for hyperuricemia by adjusted dietary n3, n6 fatty acid intake and n6: n3 ratio excluding participants with eGFR lower than 60 mL/min/1.73 m^2^ and BMI ≥ 30 kg/m^2^

| **Adjusted dietary n3 intake (mg/kg/day)** | **Case/Participants** | **Crude**  **OR (95% CI)** | **Model 1**  **OR (95% CI)** | **Model 2**  **OR (95% CI)** |
| --- | --- | --- | --- | --- |
| <14.89 | 358/2,775 | 1.00 (Ref.) | 1.00 (Ref.) | 1.00 (Ref.) |
| 14.89 to 24.48 | 462/3,910 | 0.88(0.71,1.09) | 0.87(0.70,1.07) | 0.96(0.76,1.20) |
| ≥24.48 | 476/5,006 | 0.67(0.55,0.80) | 0.64(0.53,0.77) | 0.77(0.63,0.94) |
| P for trend |  | <0.001 | <0.001 | 0.005 |
|  |  |  |  |  |
| **Adjusted dietary n6 intake**  **(mg/kg/day)** | **Case/Participants** | **Crude**  **OR (95% CI)** | **Model 1**  **OR (95% CI)** | **Model 2**  **OR (95% CI)** |
| <136.71 | 367/ 2,805 | 1.00 (Ref.) | 1.00 (Ref.) | 1.00 (Ref.) |
| 136.71 to 220.81 | 466/3,884 | 0.91(0.74,1.12) | 0.89(0.72,1.11) | 0.94(0.76,1.18) |
| ≥220.81 | 463/5,002 | 0.64(0.53,0.78) | 0.60(0.50,0.73) | 0.70(0.57,0.86) |
| P for trend |  | <0.001 | <0.001 | <0.001 |
|  |  |  |  |  |
| **Adjusted dietary n6: n3 intake** | **Case/Participants** | **Crude**  **OR (95% CI)** | **Model 1**  **OR (95% CI)** | **Model 2**  **OR (95% CI)** |
| <8.07 | 469/4,050 | 1.00 (Ref.) | 1.00 (Ref.) | 1.00 (Ref.) |
| 8.07 to 9.94 | 416/3,753 | 1.04(0.86,1.26) | 1.04(0.85,1.27) | 1.06(0.86,1.31) |
| ≥9.94 | 411/3,888 | 0.96(0.79,1.17) | 0.93(0.76,1.14) | 0.91(0.73,1.14) |
| P for trend |  | 0.61 | 0.438 | 0.338 |

Model 1 adjusted for age, gender, ethnicity.

Model 2 further adjusted for educational level, marital status, annual household income, body mass index (BMI), hypertension, smoking status, work activity, recreational activity, diabetes, total cholesterol (TC), high-density lipoprotein cholesterol (HDL-C), and total triglycerides (TG). The lowest quartile of dietary n3, n6, n6: n3 intake was used as the reference group.
