# Supplementary material for: Inverse association of dietary consumption of n3 and n6 fatty acids with hyperuricemia among adults
Source: PLoS One. 2025 Mar 13;20(3):e0317490. doi: 10.1371/journal.pone.0317490 (PMC11906074; doi:10.1371/journal.pone.0317490)
Supplement: File S1 — (ZIP) [file pone.0317490.s003.zip › Code/code.docx]

**n3_2 represents the dietary intake of n3,n6_2 represents the dietary intake of n6,n63_2 represents the ratio of dietary intake of n6/n3**

**hyper reqresents hypertension, riagendr represents gender, lbxsch represents Cholesterol, lbdhdd represents Direct HDL-Cholesterol, lbxstr represents Triglycerides, Workactivity, recreational represent the mode of physical activities.**

egen n_sbp=rownonmiss( bpxsy1 bpxsy2 bpxsy3 bpxsy4)

egen n_dbp=rownonmiss( bpxdi1 bpxdi2 bpxdi3 bpxdi4)

** Set DBP Values Of 0 To Missing For Calculating Average **

mvdecode bpxsy1-bpxsy4, mv(0)

mvdecode bpxdi1-bpxdi4, mv(0)

** Calculate Mean Systolic and Diastolic (over non-missing values) **

egen mean_sbp=rowmean(bpxsy1 bpxsy2 bpxsy3 bpxsy4)

egen mean_dbp=rowmean(bpxdi1 bpxdi2 bpxdi3 bpxdi4)

gen hyper=1 if ( (mean_sbp>=130 & !missing(mean_sbp)) | (mean_dbp >= 80 & !missing(mean_dbp)))

replace hyper = 0 if (missing(hyper) & n_sbp > 0 & n_dbp > 0)

| bpq050a == 1

capture drop age

gen age=1 if ridageyr >=20 & ridageyr <40

replace age=2 if ridageyr >=40 & ridageyr <60

replace age=3 if ridageyr >=60 & ridageyr <.

gen hyper=1 if ( (mean_sbp>=130 & !missing(mean_sbp)) | (mean_dbp >= 80 & !missing(mean_dbp))| bpq050a == 1)

replace hyper = 0 if (missing(hyper)& n_sbp > 0 & n_dbp > 0)

capture label drop sexfmt

label define sexfmt 1 "male"

label define sexfmt 2 "female", add

gen race=1 if ridreth1==3

replace race=2 if ridreth1==4

replace race=3 if ridreth1==1

replace race=4 if ridreth1==2

replace race=5 if ridreth1==5

label define racefmt 1 "Non-Hispanic White",

label define racefmt 2 "Non-Hispanic Black", add

label define racfmt 3 "Mex American", add

label define racefmt 4 "Other Hispanic", add

label define racefmt 5 "Other Race - Including Multi-Racial", add

label values race racefmt

gen edu=1 if dmdeduc2 <3

replace edu=2 if dmdeduc2==3

replace edu=3 if dmdeduc2>3 & dmdeduc2<7

gen marry=1 if (dmdmartl ==1 | dmdmartl==6)

replace marry=2 if (dmdmartl==2 | dmdmartl==3 | dmdmartl==4 | dmdmartl==5)

gen bmi2=1 if bmxbmi<30

replace bmi2=2 if bmxbmi>=30 & bmxbmi <.

gen income=1 if indhhin2 <5 | indhhin2==13

replace income=2 if indhhin2>=5 & indhhin2<=15 & indhhin2!=13

gen smoke=1 if smq020 ==1

replace smoke=2 if smq020==2

gen drink=1 if alq101 ==1

replace drink=2 if alq101==2

gen stroke=1 if mcq160f ==1

replace stroke=2 if mcq160f==2

gen bmi=1 if bmxbmi<25

replace bmi=2 if bmxbmi>=25 & bmxbmi <30

replace bmi=3 if bmxbmi>=30 & bmxbmi <.

gen diabetes=1 if diq010 ==1

replace diabetes=2 if diq010==2

gen chole=1 if lbdtcsi <=4.58

replace chole=2 if lbdtcsi>4.58 & lbdtcsi<.

gen hyperuricemia =0 if (lbxsua <6 & riagendr==2 | lbxsua < 7 & riagendr==1)

replace hyperuricemia =1 if (lbxsua >=6 &lbxsua <.& riagendr==2 ) | ( lbxsua >=7 & lbxsua <. & riagendr==1)

gen n63_2=1 if n63 <8.07

replace n63_2=2 if n63 >=8.07 & n63 <9.94

replace n63_2=3 if n63 >=9.94 & n63 <.

gen n3_2=1 if n3kg<14.89

replace n3_2=2 if n3kg>=14.89 & n3kg<24.48

replace n3_2=3 if n3kg>=24.48 & n3kg <.

gen n6_2=1 if n6kg<136.71

replace n6_2=2 if n6kg>=136.71 & n6kg<220.81

replace n6_2=3 if n6kg>=220.81 & n6kg <.

gen wtmec10yr = wtmec2yr / 5

svyset [w=wtmec10yr], psu(sdmvpsu) strata (sdmvstra)

gen n3=( dr1tp183 + dr2tp183 + dr1tp184 + dr2tp184 + dr1tp205 + dr2tp205 + dr1tp225 + dr2tp225+ dr1tp226+ dr2tp226 )/2

svyset sdmvpsu [pweight=wtmec10yr], strata(sdmvstra) vce(linearized)

svy:tabulate recreational hyperuricemia, subpop(if eligible==1) column row obs percent pearson null wald

gen active_=1 if active<500

replace active_=2 if active>=500 & active <1000

replace active_=3 if active>=1000 & active <.

gen recreational=1 if paq650==1

replace recreational=2 if (paq665==1 & recreational==.)

replace recreational=3 if recreational==.

gen Workactivity=1 if paq605==1

replace Workactivity=2 if (paq620==1 &Workactivity==.)

replace Workactivity=3 if Workactivity==.

svyset [w=wtmec10yr], psu(sdmvpsu) strata(sdmvstra) vce(linearized)

**crude model**

xi: svy, subpop(if eligible==1): logistic hyperuricemia i.n3_2

xi: svy, subpop(if eligible==1): logistic hyperuricemia i.n6_2

xi: svy, subpop(if eligible==1): logistic hyperuricemia i.n63_2

**model 2**

xi: svy, subpop(if eligible==1): logistic hyperuricemia i.n3_2 i.age i.riagendr i.race

xi: svy, subpop(if eligible==1): logistic hyperuricemia i.n6_2 i.age i.riagendr i.race

xi: svy, subpop(if eligible==1): logistic hyperuricemia i.n63_2 i.age i.riagendr i.race

**model 3**

xi: svy, subpop(if eligible==1): logistic hyperuricemia i.n3_2 i.age i.riagendr i.race i.edu i.income i.bmi i.smoke i.drink i.Workactivity i.recreational i.hyper i.diabetes lbxsch lbdhdd lbxstr i.marry

xi: svy, subpop(if eligible==1): logistic hyperuricemia i.n6_2 i.age i.riagendr i.race i.edu i.income i.bmi i.smoke i.drink i.Workactivity i.recreational i.hyper i.diabetes lbxsch lbdhdd lbxstr i.marry

xi: svy, subpop(if eligible==1): logistic hyperuricemia i.n63_2 i.age i.riagendr i.race i.edu i.income i.bmi i.smoke i.drink i.Workactivity i.recreational i.hyper i.diabetes lbxsch lbdhdd lbxstr i.marry

gen extraE=1 if ((energy <=500 | energy>=5000) & energy!=. & riagendr==2 )| ((energy <=500 | energy>=8000 & energy!=.) & riagendr==1)

svyset sdmvpsu [pweight=wtmec10yr], strata(sdmvstra) vce(linearized)

svy:tabulate riagendr hyperuricemia, subpop(if ridageyr >=20) column row obs percent pearson null wald

gen eligible=1 if wtmec2yr!=. & ridageyr>=20 & ridageyr!=. &rhq200!=1 /*

*/ & rhd143!=1 & dr1drstz==1& dr2drstz==1 & bmxwt!=. & extraE!=1 & hyperuricemia!=.

gen wtmec10yr = wtmec2yr / 5

svyset [w=wtmec10yr], psu(sdmvpsu) strata (sdmvstra)

svy:tabulate hyper hyperuricemia, subpop(if eligible==1) column row obs percent pearson null wald

xi: svy: logit hyperuricemia fibers1 fibers2 i.age i.riagendr /*

*/i.race i.edu i.income i.drink i.smoke i.bmi i.hyper /*

*/ i.diabetes lbxsch lbdhdd lbxstr i.recreational i.Workactivity

xi: svy: logit hyperuricemia fibers1 fibers2 i.age i.riagendr /*

*/i.race i.edu i.income i.drink i.smoke i.bmi i.hyper /*

*/ i.diabetes lbxsch lbdhdd lbxstr i.recreational i.Workactivity

**rcs for n3**

svyset sdmvpsu [pw = wtmec10yr], strata( sdmvstra ) singleunit(centered)

centile n3kg if eligible ==1 , centile(1,99)

drop if n3kg <= 4.08 | n3kg >= 73.16

generate n3kg1 if eligible==1 = round( n3kg )

_pctile n3kg1, p(5 50 95)

return list

mkspline fibers = n3kg1, knots(7 19 46) cubic

xi: svy, subpop(if eligible==1): logit hyperuricemia fibers1 fibers2 i.age i.riagendr /*

*/i.race i.edu i.income i.bmi i.smoke i.drink i.Workactivity i.recreational i.hyper /*

*/i.diabetes lbxsch lbdhdd lbxstr i.marry

testparm fibers2

testparm fibers2 fibers1

capture drop pa or lb ub

levelsof n3kg1, local(level)

xblc fibers1-fibers2, covname(n3kg1) at(`r(levels)') > reference(4) eform generate(pa ors lbs ubs)

twoway (line lbs ubs ors pa , sort lc(black black black) lp(- -)) , legend(off) xlabel(4(5)70) ylabel(0.1(0.1)1.1, angle(horiz) format( %2.1fc)) ytitle("Multivariate-adjusted ORs of hypertension") xtitle("Cereal fiber intake, g/day") name(f1,replace)

**rcs for n6**

svyset sdmvpsu [pw = wtmec10yr], strata( sdmvstra ) singleunit(centered)

centile n6kg if eligible ==1 , centile(1,99)

drop if n6kg <= 35.91 | n6kg >= 582.21

generate n6kg1 if eligible==1 = round( n6kg )

_pctile n6kg1, p(5 50 95)

return list

mkspline fibers = n6kg1, knots(65 176 394) cubic

xi: svy, subpop(if eligible==1): logit hyperuricemia fibers1 fibers2 i.age i.riagendr /*

*/i.race i.edu i.income i.drink i.smoke i.bmi i.hyper /*

*/ i.diabetes lbxsch lbdhdd lbxstr i.recreational i.Workactivity

testparm fibers2

testparm fibers2 fibers1

capture drop pa or lb ub

levelsof n6kg1, local(level)

xblc fibers1-fibers2, covname(n6kg1) at(`r(levels)') > reference(36) eform generate(pa ors lbs ubs)

twoway (line lbs ubs ors pa , sort lc(black black black) lp(- -)) , legend(off) xlabel(30(50)565) ylabel(0.1(0.1)1.1, angle(horiz) format( %2.1fc)) ytitle("Multivariate-adjusted ORs of hypertension") xtitle("Cereal fiber intake, g/day") name(f1,replace)
