# Supplementary material for: Inverse association of dietary consumption of n3 and n6 fatty acids with hyperuricemia among adults
Source: PLoS One. 2025 Mar 13;20(3):e0317490. doi: 10.1371/journal.pone.0317490 (PMC11906074; doi:10.1371/journal.pone.0317490)
Supplement: Attachment — Submitted filename: Response_to_Reviewers_auresp_2.docx [file pone.0317490.s006.docx]

Thank you very much for your valuable feedback. I appreciate the time and effort you have taken to review my manuscript. I am more than happy to address your questions and provide any additional information you may need.

Reviewer #2: THe authors claim that ther eis no data in the literature that they used about the action of PUFAs on xanthine oxidase activity.

I am aware of this fact.

This is the reason why I asked the authors to present preliminary evidence that PUFAs may have an action on xanthine oxidase enzyme activity. I request the authors once again to provide some evidence on this account.

Response:

Thank you very much for your suggestion! Your idea of providing evidence on how n-3 and n-6 diets might affect xanthine oxidase activity is excellent. However, your suggestion to collect data on changes in xanthine oxidase activity levels in patients following an n-3/n-6 diet is currently challenging for us. This is because our experimental data comes from the U.S. NHANES database, which lacks data on xanthine oxidase activity. Therefore, we cannot directly verify this based on the NHANES database. As a substitute, we conducted a literature search to see if there is any relevant evidence available.

After reviewing relevant English-language scientific literature, including articles indexed in PubMed and Web of Science, there appears to be no well-established, peer-reviewed evidence demonstrating that n-3 (omega-3) or n-6 (omega-6) polyunsaturated fatty acids directly inhibit xanthine oxidase activity in a manner comparable to known inhibitors such as allopurinol or febuxostat.

Most research on n-3 and n-6 fatty acids focuses on their roles in inflammation, cardiovascular health, oxidative stress modulation, and metabolic pathways rather than direct enzymatic inhibition of xanthine oxidase. Although some studies have explored the broader antioxidant properties and the indirect effects of these fatty acids on cellular redox states, they do not provide clear evidence of a direct inhibitory interaction with xanthine oxidase. For instance, reviews and primary research articles discussing dietary polyunsaturated fatty acids often highlight their impact on signaling pathways, membrane fluidity, and the modulation of inflammatory mediators (e.g., eicosanoids) rather than direct enzyme inhibition.

A keyword-based search (e.g., "omega-3 fatty acids xanthine oxidase inhibition," "omega-6 fatty acids xanthine oxidase") yields no significant, widely cited studies conclusively showing direct inhibition. Publications primarily focus on the known inhibitors of xanthine oxidase or on the physiological and biochemical implications of altered purine metabolism, rather than attributing a direct inhibitory role to n-3 or n-6 fatty acids.

In summary, based on the current available English-language academic literature, there is no strong or direct experimental evidence supporting the notion that n-3 or n-6 fatty acids function as significant inhibitors of xanthine oxidase.

We believe your suggestion is excellent. Although it is somewhat challenging for us to implement at the moment, we are enthusiastic about incorporating the investigation of the effects of n-3 and n-6 diets on xanthine oxidase activity into our future research plans as a prospective clinical trial.

Thank you again for your response and suggestions.
