# Supplementary material for: Inverse association of dietary consumption of n3 and n6 fatty acids with hyperuricemia among adults
Source: PLoS One. 2025 Mar 13;20(3):e0317490. doi: 10.1371/journal.pone.0317490 (PMC11906074; doi:10.1371/journal.pone.0317490)
Supplement: Attachment — Submitted filename: Response to Reviewers.docx [file pone.0317490.s005.docx]

Thank you very much for your valuable feedback. I appreciate the time and effort you have taken to review my manuscript. I am more than happy to address your questions and provide any additional information you may need.

Reviewer #1: The manuscript is well prepared and the topic fall into the scope of the Journal. The concern is as follows:

How did the authors to calculate the dietary consumption of n3 and n6 fatty acid? Please provide the relevant information in detail.

Response:

In the NHANES database, the dietary intake of n3 and n6 fatty acids is typically assessed using two 24-hour dietary recalls. Here are the detailed steps:

Data Collection:

Participants are asked to recall all the foods and beverages they consumed in the past 24 hours.

These recall data are collected on two different days to ensure representativeness and accuracy.

Data Processing:

The collected dietary data are entered into a standardized database.

The nutrient content of each food and beverage, including n3 and n6 fatty acids, is calculated based on the USDA’s food composition database.

Nutrient Calculation:

Using standardized food codes and nutrient composition tables, the total intake of n3 and n6 fatty acids for each participant is calculated.

These calculations take into account the type, quantity, and frequency of the foods consumed.

Data Analysis:

Through statistical analysis, researchers can evaluate the association between the intake of n3 and n6 fatty acids and health outcomes.

For example, logistic regression models might be used to assess the relationship between these fatty acids’ intake and specific health events, such as strokes

Reviewer #2: In this interesting study the authors showed that higher intake of n-3/n-6 fatty acids reduces hyperuricemia.

In this study, it would have been more appropriate a=had the authors measured the activity of xanthine oxidase enzyme before and after n-3/n-6 fatty acids in a small number of healthy or hyperuricemia subjects to know thebe potential mechanisms of action.

Is it possible that n-3/n-6 fatty acids inhibit xanthine oxidase enzyme?

Response:Thank you for your suggestions! Our manuscript is based on the general information and clinical test data (such as complete blood count) available in the NHANES database. The measurement of xanthine oxidase activity in blood is not included in the NHANES database, making it difficult to implement your suggestion in our manuscript. Additionally, current research does not provide clear evidence that n-3 and n-6 fatty acids directly inhibit xanthine oxidase activity.
